# Orexin A and orexin receptor 1 axonal traffic in dorsal roots at the CNS/PNS interface

**DOI:** 10.3389/fnins.2014.00020

**Published:** 2014-02-11

**Authors:** Damien Colas, Annalisa Manca, Jean-Dominique Delcroix, Philippe Mourrain

**Affiliations:** ^1^Department of Biology, Stanford UniversityStanford, CA, USA; ^2^Laboratory of Neurodegeneration and Axon Dynamics, European Brain Research InstituteRome, Italy; ^3^Department of Psychiatry and Behavioral Sciences, Center for Sleep Sciences, Beckman Center, Stanford UniversityStanford, CA, USA; ^4^INSERM 1024, Ecole Normale SupérieureParis, France

**Keywords:** orexin, hypocretin, receptor 1, dorsal root ganglia, axonal transport, nociception, substance P

## Abstract

Hypothalamic orexin/hypocretin neurons send long axonal projections through the dorsal spinal cord in lamina I–II of the dorsal horn (DH) at the interface with the peripheral nervous system (PNS). We show that in the DH OXA fibers colocalize with substance P (SP) positive afferents of dorsal root ganglia (DRG) neurons known to mediate sensory processing. Further, OR1 is expressed in p75^NTR^ and SP positive DRG neurons, suggesting a potential signaling pathway between orexin and DRG neurons. Interestingly, DRG sensory neurons have a distinctive bifurcating axon where one branch innervates the periphery and the other one the spinal cord (pseudo-unipolar neurons), allowing for potential functional coupling of distinct targets. We observe that OR1 is transported selectively from DRG toward the spinal cord, while OXA is accumulated retrogradely toward the DRG. We hence report a rare situation of asymmetrical neuropeptide receptor distribution between axons projected by a single neuron. These molecular and cellular data are consistent with the role of OXA/OR1 in sensory processing, including DRG neuronal modulation, and support the potential existence of an OX/HCRT circuit between CNS and PNS.

## Introduction

Orexin A and B (OXA and B also known as hypocretin 1 and 2, HCRT1 and 2) are neuropeptides produced within the lateral/perifornical hypothalamus, derived from a single prepro-peptide (de Lecea et al., 1998; Sakurai et al., 1998). While their cell bodies form a compact cluster within the hypothalamus, OX/HCRT neurons project widely throughout the central nervous system (CNS) from the olfactory bulbs to the spinal cord in both mammals and fishes (Cutler et al., 1999; van den Pol, 1999; Appelbaum et al., 2009; de Lecea, 2010). These widespread projections are matched by the expression of the two orexin receptors (OR1 and 2) (Hervieu et al., 2001; Marcus et al., 2001). OR1 displays higher affinity for OXA than OXB, whereas OR2 has equal affinity for both ligands. Orexins play a crucial role in orchestrating mechanisms related to the level of arousal such as feeding, reward-seeking, metabolism, and energy expenditure (for review see Tsujino and Sakurai, 2013). This neuropeptidergic system is particularly well known for stabilizing the sleep-wake states and is responsible for narcolepsy when disrupted (Peyron et al., 2000; Sakurai, 2013; Sorensen et al., 2013). As such, OX innervations of other sleep-regulating brain nuclei are extensively studied. While it is well known that OX axons project throughout the dorsal spinal cord (Cutler et al., 1999; van den Pol, 1999; Appelbaum et al., 2009; de Lecea, 2010), the interaction of the central OX system with the peripheral nervous system (PNS) including the dorsal root ganglia (DRG) (Hervieu et al., 2001) has been less explored. Interestingly, several studies have shown that OXA and OR1 can modulate sensory and nociception processing (for review Chiou et al., 2010).

**Figure 1:**
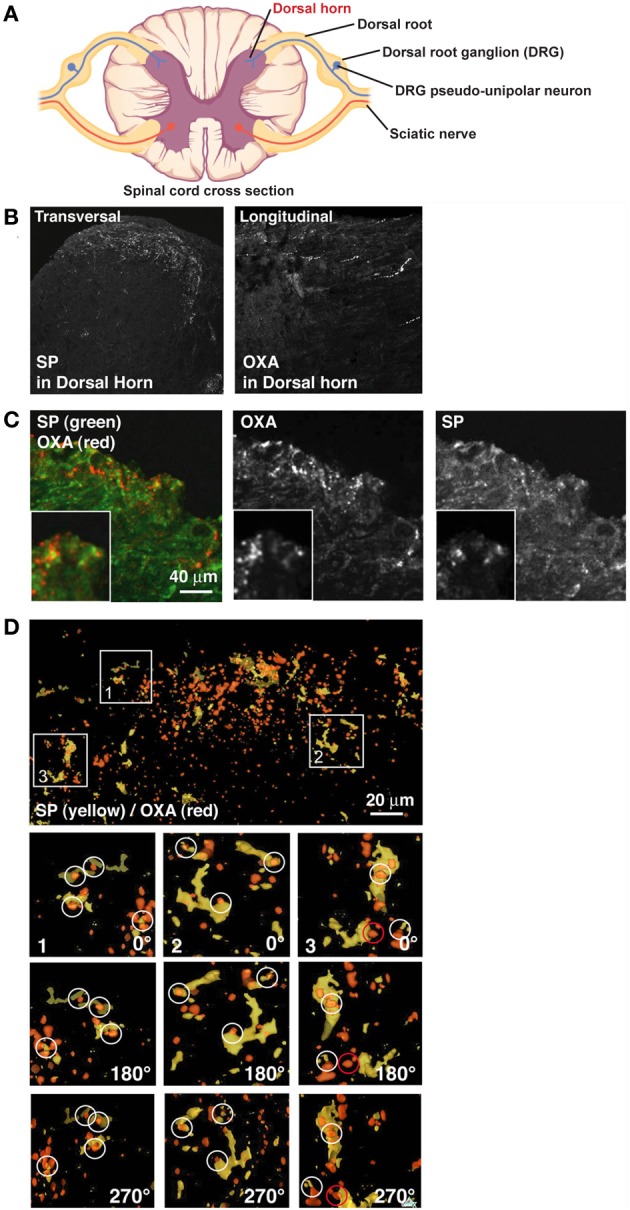
**OXA hypothalamic fibers appose with SP positive DRG fibers in the dorsal horn**. **(A)** Scheme of a spinal cord cross section (modified from OpenStax College PNS course). Dorsal horn, site of analyses presented below, is indicated in red. **(B)** Left panel: transversal section of spinal cord stained for SP. SP decorates lamina I and II of the dorsal horn. Right panel: longitudinal section of spinal cord stained for OXA. OXA is present in lamina I and II of the dorsal horn. **(C)** Transversal section of lamina I and II of the dorsal horn stained for OXA (red) and SP (green). **(D)** While some colocalization was visible in B, juxtaposition became clear only after a 3D reconstruction (SP in yellow and OXA in red). In inserts 1, 2, and 3, SP terminals in the section were colocalized or in contact with OXA terminals. Inserts were rotated by 180° (first panel under the insert) and by 270° (second panel under the insert). White circles show apposition of SP and OXA and a red circle shows an example of false positive.

DRG neurons are pseudo-unipolar neurons that have the unique property of projecting axon branches toward the spinal cord and toward peripheral targets (Figure 1A) (Kandel et al., 2000). In the periphery, DRG neurons innervate the skin and muscle (Kandel et al., 2000). In the spinal cord, afferents of proprioceptive DRG neurons terminate in the ventral horn, afferents of cutaneous mechanoreceptive DRG neurons terminate in the deep dorsal horn (DH) and afferents of nociceptive and thermoreceptive DRG neurons terminate in the superficial (lamina I/II) DH (Kandel et al., 2000). The difference between projection areas of DRG neurons in the central and the PNS prompted us to question whether neurotransmission material present in both axon branches was identical (Delcroix et al., 2004). Here, we show that in the DH of the spinal cord lamina I and II, DRG afferents closely appose with the OXA descending innervation arising from the hypothalamus. We also observed that OR1 is expressed in DRG neuron cell bodies and detected in the dorsal root (DR) connecting the DRG to the spinal cord but not in the sciatic nerve (SN). This data suggests that the anterograde axonal transport of OR1 can be directed toward one axon branch and not the other. Consistent with this finding, OXA is also detected in the DRG neuron cell bodies suggesting a retrograde transport from the spinal cord to the DRG. This body of work supports at the cellular level the existence of an interaction between the central hypothalamic orexin neurons and the PNS. Understanding the influence of orexins on the PNS physiology is not only important for its function in arousal threshold changes but also potentially relevant to OXA anti-nociceptive function (Dauvilliers et al., 2011; Doghramji, 2012; Roehrs et al., 2012).

## Results

### Orexin a axonal fibers and SP positive fibers from DRG are juxtaposed in spinal cord lamina I and II

CNS OX/HCRT neurons are well known for their long axonal projections to the dorsal spinal cord (Cutler et al., 1999; van den Pol, 1999; Appelbaum et al., 2009). Similarly, PNS axons coming from the DRG and releasing Substance P (SP) connect the CNS in lamina I and II of the spinal cord DH (Nichols et al., 1999), suggesting a possible interaction with the OX/HCRT circuit in these layers. Thus, we first inquired if afferents of SP neurons in the spinal cord were in physical contact with OXA-positive fibers arising from the hypothalamus. Axons expressing OXA propagate into lamina I and II by spreading longitudinally (Figure 1B) (Cutler et al., 1999; van den Pol, 1999) whereas the SP positive fibers branch and spread robustly in a coronal fashion in these layers (Figure 1B). OXA and SP double immunofluorescent histochemistry staining suggested colocalization of these fibers (Figure 1C), but due to these innervation orthogonal characteristics it was difficult to analyze the extent of the apposition between SP and OXA either in coronal sections or in longitudinal sections. 3D reconstructions of coronal sections (see Figure 1D) clearly showed that OXA and SP terminals are juxtaposed in lamina I and II (Figure 1D). We found that 22 ± 4% (from 4 rats, 2 sections taken from the DH) of OXA terminals colocalized with SP terminals, whereas 75 ± 6% (from 4 rats, 2 sections taken from the DH) of SP terminals colocalized with OXA terminals.

### Orexin receptor 1 is expressed in dorsal root ganglia p75^NTR^/SP neurons

**Figure 2:**
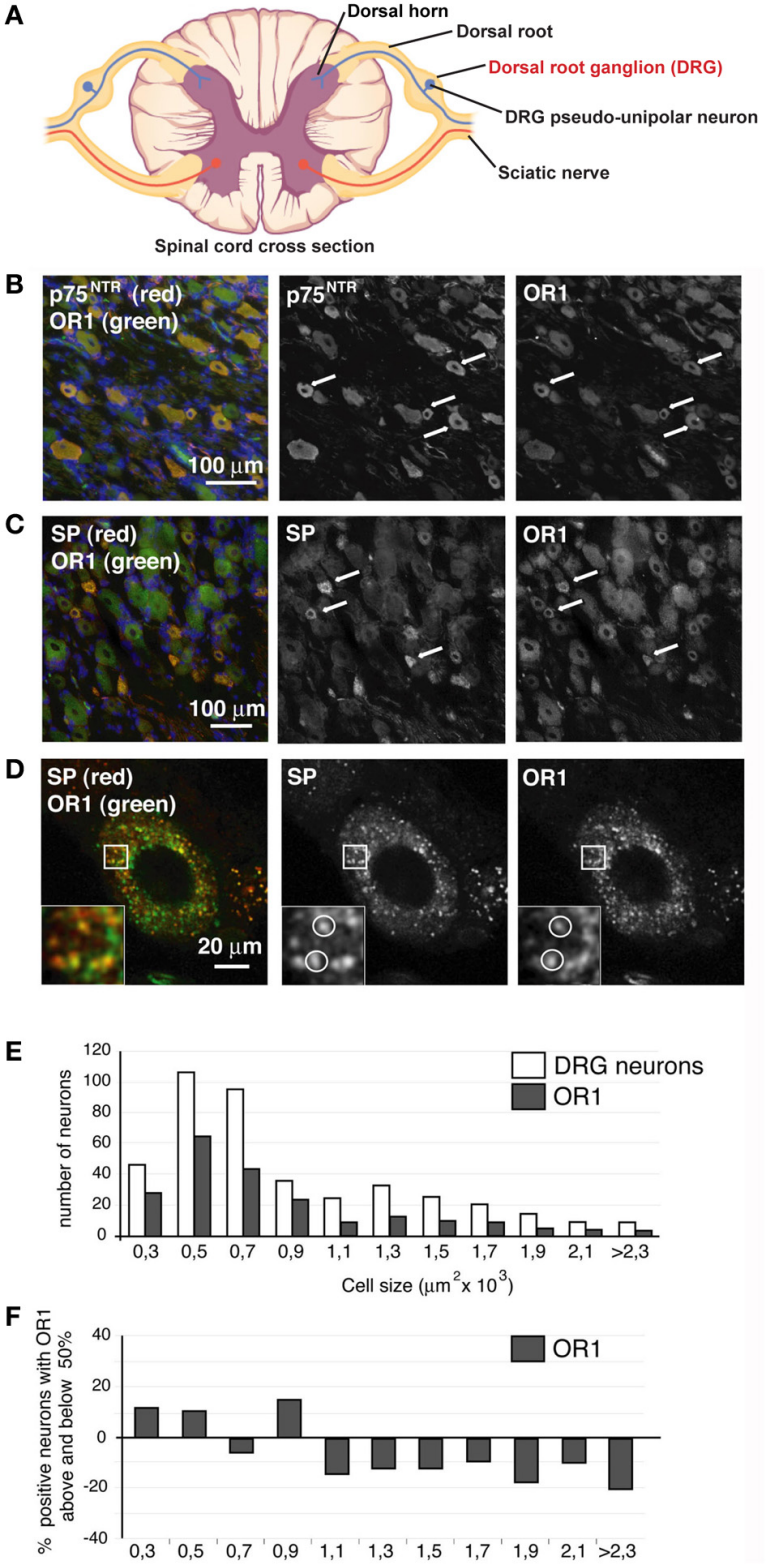
**OR1 is present in DRG neurons**. **(A)** Scheme. DRG, site of analyses presented below, is indicated in red. **(B)** DRG neurons stained for p75^NTR^ (red) and OR1 (green), nuclei were stained in blue with DAPI. Most p75^NTR^ neurons were OR1 positive (arrows). **(C)** DRG neurons stained for SP (red) and OR1 (green). All SP positive neurons were strongly positive for OR1. **(D)** SP (red) and OR1 (green) showed a punctuated pattern as well as a clear colocalization in a third of SP puncta. **(E)** Size distribution of OR1 in DRGs compared to the size distribution of all DRG neurons. **(F)** Percentage of DRG neurons of a given size positive for OR1 (the numbers are given as percentage above and below 50%; baseline 0 corresponds to 50% of neurons positive for OR1). Arrows and white circles denote colocalization.

Since the OXA positive terminals were potentially in contact with SP positive fibers, we tested if OR1 was present in DRG neurons (see schematic Figure 2A) expressing SP or p75 neurotrophin receptor (p75^NTR^), another marker for sensory neurons (Delcroix et al., 1998, 2003) (Figure 2B). Staining for p75^NTR^ and OR1 was neuronal since the nuclei of satellite cells stained with DAPI did not show OR1 staining (Figure 2B). The percentage of p75^NTR^-positive cells was 43 ± 4% (from 4 rats, 2 sections taken from the middle of the DRG). The percentage of OR1-positive cells was 57 ± 5% (from 4 rats, 2 sections taken from the middle of the DRG). The neuroanatomical analysis showed that p75^NTR^-positive neurons were predominantly small and medium sized neurons (cell bodies ranging from 250 to 750 μm^2^, Figure 2E). The size of OR1-positive neurons was also small and medium (Figures 2E,F). This size and morphology distribution was consistent with our previous characterization of the DRG p75^NTR^ and trkA-expressing sensory neurons (Delcroix et al., 1998, 2003).

Importantly, double staining of DRGs shows that most, if not all, of the p75^NTR^-positive neurons express OR1 (Figure 2B), even if it was also clear that neurons other than p75^NTR^-positive neurons were OR1 positive. DRG sections were also immuno-stained for OR1 and SP (Figure 2C) and we observed that all SP positive cells also expressed OR1 (Figure 2C). The large co-expression of OR1 with p75^NTR^ and SP markers hence further supports a potential interaction between the OXA terminals and the DRG neurons. Higher magnification analysis of SP and OR1 double staining showed a robust punctate pattern in neuron cell bodies (Figure 2D). 32 ± 6% (*n* = 10 neurons in 4 different animals) of OR1 positive puncta in those cells colocalized with SP. Because of the secreted nature of the SP neuropeptide, the cytoplasmic colocalization of OR1 with SP prompted us to investigate whether OR1 expressed in DRG neurons was present in the trafficking pathway that sends material toward nerve terminals.

### OR1 is anterogradely accumulated from DRG to the spinal cord while OXA is retrogradely accumulated from the spinal cord to the DRG

**Figure 3:**
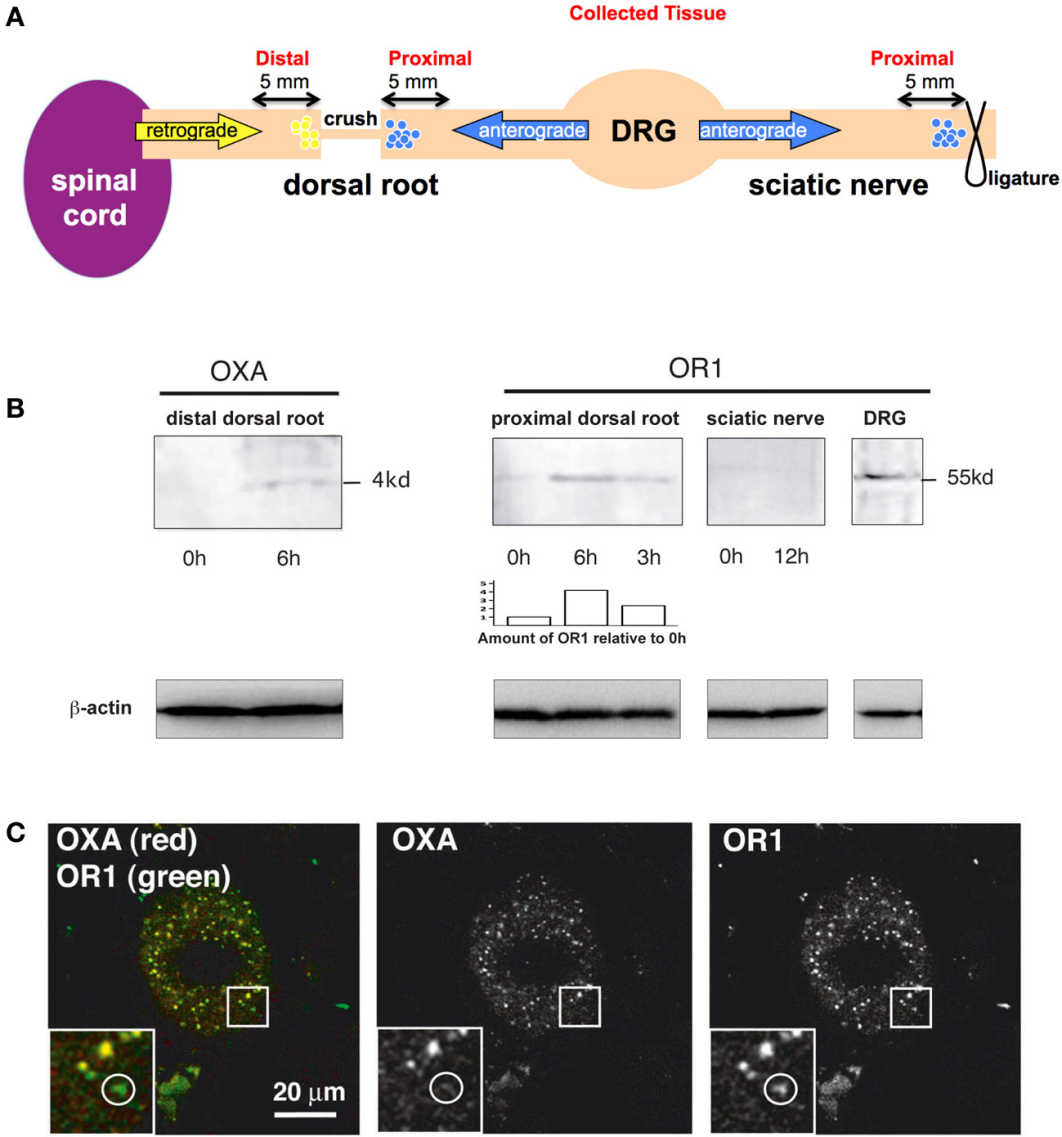
**OR1 and OXA are transported in dorsal root axons**. **(A)** Scheme illustrating the crush and ligature experiments and indicating the sites where tissue samples were collected to measure OXA and OR1 accumulation. **(B)** Transport analysis (based on our previous work Delcroix et al., 2003). Left panel, OXA is accumulated after 6 h in the dorsal root suggesting a retrograde transport. Right panel, 6 and 3 h OR1 accumulation proximal at a crush site in the dorsal roots. The 0 h lane has been loaded with an intact root from the same animal. Blot analysis and accumulation of OR1 was determined using ImageJ and normalized to the 0 h value (see bar chart). Note the progressive accumulation over time of OR1 suggesting an anterograde transport. 12 h accumulation in the SN did not show OR1 accumulation. DRG tissue was used as positive control. Lower panels, β-actin was used as an internal loading control. **(C)** OXA (red) was present in the DRG and always colocalized with OR1 (green) whereas OR1 could be found without OXA (see white circle).

Since the OXA positive terminals colocalize with SP positive fibers in the spinal cord and OR1 is expressed in DRG neurons, we then tested whether OR1 was anterogradely accumulated/transported from the DRG to the spinal cord (see principle in Figure 3A). We used the nerve crush method and axonal transport analysis described in our previous works on nerve growth factor effects on sensory neurons (Delcroix et al., 1998, 2003). We performed laminectomies (bone removal allowing access to DRG and DR) on rats and crushed the dorsal roots (DRs) arising from lumbar 4 and 5 (L_4_ and L_5_) DRGs (Figure 3A). Nerve crush leads to accumulation of normally transported material on both sides of the crush (Figure 3A). The DRs were collected at 3 time points, 0, 3, and 6 h (3 animals were used for each time point), and the proximal section of the crushed DR was run on an SDS-Page gel (Figure 3B, right panel). OR1 accumulated over time (Figure 3B, right panel). As a control, DRG was also tested and as expected, OR1 was expressed in the ganglia. In striking contrast, OR1 was not accumulated in the SN toward the periphery even after 12 h (Figure 3B, right panel). We do not know whether it involves a passive or active transport, however this data clearly suggests that OR1 is selectively accumulated toward the spinal cord and not the periphery. This data further strengthens the possibility of an orexin/hypocretin neurotransmission between the CNS and PNS/DRG. More importantly, from a more general neurobiological perspective, this observation is also a rare case of asymmetrical neuropeptide receptor distribution between axons projected by a single neuronal cell type.

We next tested whether OXA secreted by hypothalamus-originating fiber terminals could be taken up by afferents arising from the DRG. We looked for retrogradely transported OXA by withdrawing material accumulated at the crush site of DRs arising from L_4_ and L_5_ DRGs (retrograde accumulation was harvested after 6 h) (Figure 3B, left panel). OXA accumulated at the crush site (*n* = 3 animals) as opposed to intact DRs (0 h transport) (Figure 3B, left panel). Furthermore, we found that retrograde transport of OXA was specific, as it was present in DRG neuron cell bodies always colocalizing with its receptor (Figure 3C). Altogether these results suggest the existence of neurotransmission between the central OX/HCRT neurons and the peripheral SP neurons. The existence of a functional circuit between OX/HCRT and DRG neurons is yet to be fully demonstrated.

## Discussion

The discovery of molecular motors, and in particular kinesin, has led to the realization that cell trafficking was a highly controlled phenomenon (Vale et al., 1985). Despite our present extended knowledge of cellular trafficking, our comprehension of transport events in neurons is incomplete. Moreover, the possibility of transport directed toward specific axon branches poses a clear challenge to our understanding of axonal transport toward specific targets. In this work we show that indeed a specific protein, OR1, can be transported—via a mechanism yet to be deciphered—in one axon branch (DR) but not in the other (SN). Much evidence points to the existence of changes in transport flux in axon branches. Goldberg and Schacher showed that cultured *Aplysia californica* cerebral neurons grow from both ends of both branches (Goldberg and Schacher, 1987). When a branch was next to a target cell, bidirectional fast axonal transport of organelles was more robust in that branch. A similar observation was made in hippocampal neurons by Ruthel and Hollenbeck (2003). Indirect evidence also pointed to differential axonal transport *in vivo*. Schreyer and Skene showed that injuries of the SN or the DR did have different effects in DRG neurons, implying the existence of a different retrograde signal that might be linked to a different composition in the proteins anterogradely transported in both axon branches (Schreyer and Skene, 1993). Differential transport in axon branches might also be linked to morphological changes occurring in network rearrangements during embryonic and adult life (for review see Luo and O'Leary, 2005). It is unclear what mechanism mediates differential transport in axon branches and it is beyond the scope of this study. Nevertheless, in this work we link differential OR1 accumulation to an OXA/SP network that only exists in the spinal cord and not in the periphery.

OXA was retrogradely accumulated from the spinal cord to the DRG and was always detected in the DRG in the presence of its receptor. The potential retrograde transport of a receptor-bound neuropeptide may be an important process, which conveys information from the synapse to the DRG cell bodies. OXA could also play a role in the regulation of cell mechanisms as shown for neuropeptide Y that regulates axonal transport of organelles in neurites of cultured DRGs (Hiruma et al., 2002). In addition, OR1 is a G protein-coupled receptor (GPCR). Its binding to OXA and potential subsequent internalization in SP-positive synapses might have a similar role to the one played by the internalization of other DRG GPCRs. For instance, the opioid receptor-like receptor controls the availability of calcium channels through internalization and consequently modulates long-term pain related signaling (Altier et al., 2006).

We do not demonstrate here the existence of a functional circuit between central OX/HCRT neurons and peripheral DRG neurons, however we bring evidence suggesting a potential interaction between these neurons. While we do not show synaptic connections, the paracrine release of OX/HCRT neuropeptides in close proximity to DRG neuron fibers expressing OR1 could suffice for neurotransmission. This hypothesis is substantiated by the axon-specific accumulation of OR1 in DRG pseudo-unipolar neurons and the presence of its ligand OXA in the cell bodies. We believe that the presence of OXA neuropeptide in the DRG neurons is due to its uptake and not to its cell-autonomous expression in these neurons. Besides the lateral hypothalamus, OX/HCRT mRNA has indeed never been detected in the nervous system elsewhere in all species studied so far, including fishes (zebrafish Faraco et al., 2006; Appelbaum et al., 2009, goldfish Nakamachi et al., 2006), birds (chicken Ohkubo et al., 2002, quail Phillips-Singh et al., 2003), and mammals (de Lecea et al., 1998; Sakurai et al., 1998 and Luis de Lecea pers. commun.). Of course, we cannot rule out a sub-threshold expression of OX/HCRT transcript in the DRG, but it seems less likely compared to its uptake in the dorsal spinal cord.

Finally, a potential network between the CNS and PNS may account for the involvement of the OX/HCRT in the modulation of sensorial processing. An increasing body of knowledge shows that the orexin system is involved in pain regulation and orexin peptides exert antinociceptive actions (Bingham et al., 2001; Holland et al., 2005, 2006; Kajiyama et al., 2005; Mobarakeh et al., 2005; Yan et al., 2008; Ho et al., 2011; Feng et al., 2012) and for review Chiou et al. (2010). In particular, OXA decreases nociception mostly via OR1 both at spinal and supra-spinal levels in various models of pain. For instance, in an acute model of pain (hot plate test), intrathecal injections of OXA proved to be anti-nociceptive (Yamamoto et al., 2002). In addition, in a model of chronic neuropathic pain (chronic constrictive injury of SN), Jeong and Holden showed that stimulation of posterior hypothalamus induces anti-nociception and that the anti-nociceptive effect is mediated in part by the OR-1 receptor in the spinal cord DH. Their findings, in combination with our findings suggest a possible role of axonal transport of OR-1 and OXA in pain models of chronic constrictive nerve injuries (Jeong and Holden, 2009). Cellular effects of orexins on pain mechanisms have scarcely been studied so far, but it has been shown that orexins modulate the electrophysiological properties of key pain modulatory centers, such as the spinal cord DH and the ventro-lateral peri-aqueductal gray vlPAG (Grudt et al., 2002; Ho et al., 2011). Further, the anti-nociceptive effects of OXA were endocannabinoid dependent in the vlPAG (Ho et al., 2011) and endoncannabinoid independent in the DH (Bingham et al., 2001). Moreover, orexin mediates the analgesia in the model of stress-induced analgesia (Xie et al., 2008; Gerashchenko et al., 2011). Surprisingly, in some instances anti-nociceptive effects of peripheral OXA injections have also been shown (Bingham et al., 2001; Holland et al., 2005, 2006). Importantly, patients suffering from narcolepsy with cataplexy, a condition associated with a disrupted OX/HCRT system, experience a higher frequency of pain (Dauvilliers et al., 2011). Anatomical and cellular studies may account for those peripheral effects as OR1 and OXA have indeed been found in DRG (Bingham et al., 2001) and OXA directly modulates the DRG neuronal activity (Yan et al., 2008).

Our data also allow us to speculate on the mechanism underlying the possible analgesic effects of peripheral OXA injections. Indeed, OXA might affect SP-positive DRG neurons by binding to OR1, thus changing the DRG-mediated nociceptive action and/or chronically adapting the pain sensation threshold. Such findings may have practical importance in the wake of current hypnotic drug development aiming at blocking orexin receptors using peripheral routes (Scammell and Winrow, 2011).

## Materials and methods

### Animals

Adult (6-month-old) male Wistar rats (300–350 g) were purchased from Harlan (Italy), and all experiments were performed according to the national and international laws for laboratory animal welfare and experimentation (EEC council directive 86/609, 12 December 1987) under a license issued by the Local Animal Ethics Committee. Rats were kept under a 12-h dark–light cycle, at 23 ± 1°C, with food and water *ad libitum*.

### Surgery

Rats were anesthetized with chloral hydrate at 500 mg/kg and subjected to a laminectomy. Axonal transport was measured in DRs 3 or 6 h after the application of crushes on the right side of the animal (Raivich et al., 1991; Delcroix et al., 1997) (*Nota bene*: because of the thin nature of the DR tissue, crush was favored over ligature). Crushes were made with watchmaker's forceps, with compression for a period of 30 s and repeated twice as in our previous work (Delcroix et al., 1997, 2003). After the laminectomy, the wound was closed with Michel clips and the animal was monitored for recovery. Axonal transport was measured in the SN by placement of 2 tight ligatures using surgical silk for 12 h at mid thigh as described elsewhere (Raivich et al., 1991; Delcroix et al., 1997). At the end of the procedure 5 mm of DRs was harvested on both sides of the crush, proximal and distal to the DRG. Five millimeter of SN was harvested on the side of the ligature proximal to DRG (see schematic Figure 3A).

### Homogenization and western blotting

A similar volume of tissue samples from the DRs, SN, and lumbar 4 and lumbar 5 (L_4_ and L_5_) DRGs was homogenized as described previously (Filliatreau et al., 1988; Delcroix et al., 2003) using a polytron (Kinematica, Luzern, Switzerland) in 200 μl of protein extraction buffer [0.1 mM PBS (pH 7.4), 1.0 mM of NaF, 1.0 mM NaVO_4_, 2.0 mM EDTA, 0.5% Triton X-100, 1.0 mM phenyl methylsulphonyl fluoride (PMSF), and 10 μg/ml aprotinin]. Total protein content was determined using Bio-Rad protein assay (Bradford method). SDS-Page gels were then loaded with the same amount of material proportionally to 5 mm of collected sample [10 μg (~20 μl) of total proteins from DR, SN, and DRG tissues]. The separated proteins were transferred to PVDF (Millipore, Billerica, Massachusetts, USA) using a Biorad Mini Trans-blot (30 V overnight, at 4°C). The blots were incubated with a 1:200 dilution of OR1 (Santa Cruz antibodies #SC-8073, Santa Cruz, California, USA) goat polyclonal antibody or with a 1:200 dilution of OXA (Phoenix peptide #H-003-30, Belmont, California) rabbit polyclonal antibody (Santa Cruz antibodies, Santa Cruz, California, USA). Blots were then incubated with the secondary antibody (anti-rabbit HRP conjugate or anti-goat HRP conjugate from Jackson Immunoresearch Europe, UK) at 1:2000 for 60 min at room temperature. Detection was achieved using a Bio-rad Versadoc image analyzer (Bio-rad, Hercules, California, USA). Band signal quantification was achieved using ImageJ (NIH, Bethesda). Monoclonal mouse anti-actin antibody (Sigma Aldrich) was then used as a loading control for western blot analysis of proteins from rat DRG, SN, and DR.

### Immunocytochemistry

Wistar rats (6 months old) were prepared for immunohistochemical analysis using standard techniques. Anesthesia was induced with chloral hydrate (700 mg/kg); animals were perfused through the ascending aorta with saline followed by 300 ml of 4% paraformaldehyde in 0.1 M phosphate buffer, pH 7.4. L_4_ and L_5_ DRGs and the lumbar spinal cord were removed, postfixes overnight at 4°C in the same fixative and then cryoprotected in 30% sucrose overnight. DRGs and spinal cord were frozen and sectioned with a thickness of 10 μm. Sections were stained using indirect immunofluorescence histochemistry with a rabbit polyclonal antibody directed against OXA (Phoenix peptide #H-003-30, Belmont, California) diluted at 1:250, a rabbit polyclonal antibody against p75^NTR^ (#1405-41-0 Promega, Madison, Wisconsin, USA) diluted at 1:250, a mouse monoclonal antibody against SP (abcam #14184-50, Cambridge, UK) diluted at 1:250, a goat polyclonal antibody directed against OR1 at 1:250 (Santa Cruz antibodies #SC-8073, Santa Cruz, California, USA). For detection of primary polyclonal antibodies, a donkey anti-rabbit or a donkey anti-goat Cy3 or Cy2 conjugated secondary antiserum (1:250; Jackson Immunoresearch, Cambridge, UK) was used; for detection of monoclonal antibodies, a donkey anti-mouse Cy3 or Cy2 conjugate was used (Jackson Immunoresearch). Double labeling of SP together with OR1 was achieved using standard indirect immunofluorescence as described elsewhere (Merighi and Carmignoto, 2002). After final washes in phosphate buffered saline (PBS), sections were coverslipped in a PBS/glycerol solution (1:3) containing 2.5% 1,4 diazobicyclo (2,2,2) octane (antifading agent; Sigma). Immunoreactivity was visualized on a Nikon 90i epifluorescence microscope linked to a C1 confocal system.

3D reconstructions as shown in Figure 1 were produced using the software Osirix, a freeware used for tomography (http://homepage.mac.com/rossetantoine/osirix/Index2.html). Twenty optical sections were taken every 0.4 μm and were then reconstructed in 3D.

### Cell quantification

Cell size and cell density was calculated with ImageJ (NIH, Bethesda). Cell size and staining intensity were quantified when a clear nuclei was visible in the image. To decide if a cell was p75^NTR^ or OR1 positive, the following criteria was applied: an average background was obtained by quantifying the average intensity of 5 fields that did not show any staining in the image. Then, if the intensity measured for a given neuron was 3 fold above background, the cell was considered positive for p75^NTR^ or OR1. For OXA and for p75^NTR^ we quantified from 4 rats 2 sections taken from the middle of the DRG. Numerical data are expressed as mean ± standard deviation.

### Conflict of interest statement

The authors declare that the research was conducted in the absence of any commercial or financial relationships that could be construed as a potential conflict of interest.
